# Pressure Driven Rapid Reconfigurable Liquid Metal Patterning

**DOI:** 10.3390/mi14040717

**Published:** 2023-03-23

**Authors:** Bingxin Liu, Peng Qin, Mingyang Liu, Wei Liu, Pan Zhang, Zi Ye, Zhongshan Deng, Zhenming Li, Lin Gui

**Affiliations:** 1 CAS Key Laboratory of Cryogenics, Technical Institute of Physics and Chemistry, Chinese Academy of Sciences, Beijing 100190, China; 2 School of Future Technology, University of Chinese Academy of Sciences, Beijing 100049, China; 3 Energy Storage and Novel Technology of Electrical Engineering Department, China Electric Power Research Institute, Beijing 100192, China

**Keywords:** a method for liquid metal patterning, microchannels, control liquid metal, reconfigurable antennas

## Abstract

This paper proposes a method for pressure driven rapid reconfigurable liquid metal patterning. A sandwich structure of “pattern—film—cavity” is designed to complete this function. Both sides of the highly elastic polymer film are bonded with two PDMS slabs. One PDMS slab has microchannels patterned on the surface. The other PDMS slab has a large cavity on its surface for liquid metal storage. These two PDMS slabs are bonded together, face to face, with the polymer film in the middle. In order to control the distribution of the liquid metal in the microfluidic chip, the elastic film will deform under the high pressure of the working medium in the microchannels and then extrude the liquid metal into different patterns in the cavity. This paper studies the factors of liquid metal patterning in detail, including external control conditions, such as the type and pressure of the working medium and the critical dimensions of the chip structure. Moreover, both a single-pattern and a double-pattern chip are fabricated in this paper, which can form or reconfigure the liquid metal pattern within 800 ms. Based on the above methods, reconfigurable antennas of two frequencies are designed and fabricated. Meanwhile, their performance is simulated and tested by simulation and vector network tests. The operating frequencies of the two antennas are respectively significantly switching between 4.66 GHz and 9.97 GHz.

## 1. Introduction

With the development of advanced microfabrication technologies, flexible microfluidic devices have become an area of interest for various applications [1,2,3,4,5]. However, it is difficult to apply in electronics. Materials used in traditional electronic devices are almost always rigid, including metals, insulators, and semiconductors. These conventional materials will mechanically mismatch under 1–3% strain, which is a challenge for flexible microfluidic electronics [6]. Gallium and several gallium-based liquid metal alloys are liquid around room temperature, which has the properties of both liquids and metals, similar to mercury. Eutectic Ga-In alloy (75.5% gallium, 24.5% indium) is an example. As ‘liquid’, EGaIn has a low viscosity ($0.32{\;\mathrm{mm}}^{2}/s$ of EGaIn and $1{\;\mathrm{mm}}^{2}/s$ of water) and a high surface tension (0.624 N/m of EGaIn and 0.72 N/m of water), while as ‘metal’, it has high electrical conductivity ($3.4\times 10^{6}\;S/m$ of EGaIn and $72\times 10^{-3}\;S/m$ of water). Furthermore, unlike mercury, EGaIn has low toxicity and negligible vapour pressure [7].

Such alloys make ‘soft’ electronic devices possible; moreover, the ability to pattern liquid metals is the basis for fabricating metallic components that are soft and flexible in electronic devices. Over the years, an enormous amount of research has been carried out in an attempt to pattern liquid metals. These patterning methods can be divided into three categories: subtractive, additive, and injection methods [8]. Subtractive methods include imprinting [9], masked deposition [10], selective surface wetting [11], direct laser patterning [12], and so on. Gozen et al. firmly pressed an array of microchannels arranged on the PDMS surface on a layer of liquid metal film coated on the PDMS substrate [9]. Under the effect of pressure and the inherent high-viscosity oxide layer on the liquid metal surface, the liquid metal tightly filled the channel, and a liquid metal linear array with a line width of 2 microns and a spacing of 1 micron was formed. Due to the inherent characteristics of the material, PDMS has a limited pressure endurance, so this method is challenging to produce high aspect ratio patterns. At the same time, these subtractive methods will cause significant material waste.

The additive patterning method is mainly a method of liquid metal printing based on the high adhesion of the oxide layer on the surface of liquid metal, including inkjet printing, stencil printing, direct writing technology of liquid metal, and liquid metal 3D printing [13,14,15,16,17,18]. Boley et al. applied the printing method to draw the liquid metal circuit on the surface of PDMS, and produced the strain sensor based on this method [19]. These liquid metal additive patterning methods can realize the full application of liquid metal materials and automatic processing. Each of those methods has demonstrated rather distinctive advantages and showed their tremendous potential for many frontier electronics researchers. However, among those methods, it is the native oxide shells on the surface of liquid metals that allow the metal to adhere to and be shaped on surfaces. Consequently, once the liquid metal is patterned on the substrate, it will lose the property of “liquid” to some extent.

The injection method is one of the most commonly utilized patterning methods in the field of liquid metal microfluidics [20,21,22,23,24,25,26,27]. The liquid metal is injected into the microchannels embedded in the substrate by using a syringe and rapidly fills the channel according to the channel distribution, forming various patterns. Huang et al. produced a reversible stretching microfluidic serpentine antenna by the microfluidic injection method [28]. The principle of the vacuum injection method is similar to that of the microfluidic injection method. The microfluidic chip is put into a vacuum box. There is a pressure difference between the microchannel inside the chip, and the channel causes the liquid metal to fill it. Lin et al. could fill the serpentine microchannel up to several meters using this method [29]. Wang et al. proposed a porous-membrane-enabled fast liquid metal patterning method and used this method to fill several tree-shaped complex structure electrodes with blind ends in a thin chip [30]. Ye et al. adopted a femtosecond laser to drill small holes at the end of the microchannel, and the liquid metal could not escape from the small holes under the restriction of surface tension, while the air in the microchannel could escape during the metal filling process [31]. Using this technology, Ye et al. integrated the double-layer liquid metal pattern into the 119 mm thin PDMS and made a skin sensor.

In order to give full play to the fluidity of liquid metal, it is particularly important to carry out research on the reconfiguring method of liquid metal patterning. The driving principles of liquid metal manipulation and patterning reconfiguring can be summarized as external force drive, electromagnetic control, chemical drive, and self-drive [32,33,34]. More specifically, it includes mechanical stretching, bending, twisting, injection drive, and electric wetting drive. Qin et al. proposed a liquid metal microstrip patch antenna, and the liquid metal injected into the antenna can flexibly flow and deform at different rotation angles due to gravity to achieve different working states with reconfigurable frequency [35]. However, the oxide skin adhering to channel walls will form a residue on the walls, which impedes liquid metal reconfiguring in the channels. For the better realization of liquid metal reconfiguring, solutions include the use of acid, lip layers, and electrochemistry. Gong et al. proposed a method to reconfigure liquid metal in a line channel using pressure, which enabled a microvalve to acquire a high repeatability of 145 times [36]. Xu et al. developed a reconfigured antenna using a syringe to inject or withdraw liquid metal into different patterned units [37]. These methods can realize patterning and reconfiguring liquid metal, but they mainly change the shape of liquid metal in a one-dimensional scale or limited channels.

Based on microfluidic technology, this paper proposes a patterning and rapid reconfiguring scheme to realize large-area patterning and rapid reconfiguring of liquid metal on a two-dimensional scale through pressure driving. In order to evaluate the feasibility and performance of the method, this paper explores the influence of control conditions and chip structure on the patterning results and presents the switching of array patterns. To illustrate the practical value of this method, it also develops and demonstrates a group of reconfigurable antennas, which can quickly switch between the two forms with significant changes in operating frequencies. The processing technology of the currently proposed method is simple and fast, and the liquid metal patterning and reconfiguring could be realized through an easy operation. It demonstrates an excellent, flexible, reconfigurable antenna fabricating method, which can be compatible with wearable devices and flexible sensors, providing more possibilities for realizing highly integrated microfluidic flexible electronic devices.

## 2. Working Mechanism

**Figure 1:**
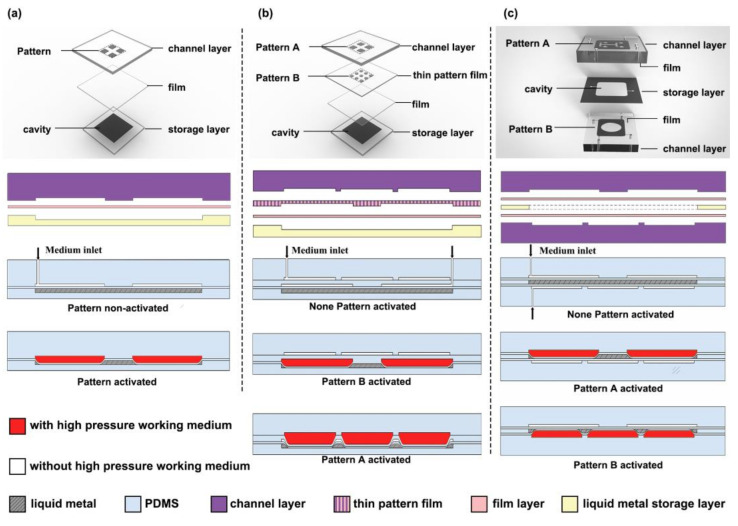
Illustration of structure and working principle of (**a**) the single-pattern chip, (**b**) the double-pattern chip, and (**c**) the reconfigurable antennas.

In order to control the liquid metal to form the specified pattern in the microfluidic chip, as shown in Figure 1a, it needs to contain three layers: a channel layer, a film, and a storage layer. The channel layer is composed of the target pattern in which the pattern of the microchannel groove is reversed in phase compared with the liquid metal target pattern. The deformable high-elastic polymer film is bonded upon the groove surface of the channel layer and can seal the groove. Furthermore, a liquid metal storage layer with a cavity is bonded with the polymer film on the other side. A high-pressure working medium is introduced to the channel through the medium inlet. The deformable film is then driven by the pressure to squeeze the liquid metal in the cavity on the other side to form the target pattern. After the pressure is removed, the elastic film can be restored to its original state, and the liquid metal pattern will disappear. When the channel layer contains multiple groups of channels, the liquid metal pattern in the cavity can be reconfigured on the two-dimensional plane by introducing a high-pressure working medium into different channels. Two holes with a diameter of 2 mm were punched for injection convenience concerning the liquid metal storage layer. Through these two holes, the cavity is connected to an external liquid metal pool and kept at atmospheric pressure. The liquid metal inside the cavity can move freely through the holes under the compression of the film.

Based on the above-mentioned single-pattern chip, the liquid metal patterning and reconfiguring in a “three-dimensional” sense is proposed through optimizing and upgrading the fabrication of elastic film, which realizes multi-layer liquid metal patterning and reconfiguring on a plane. Figure 1b shows the structure and working principle of a double-pattern chip that contains four layers. Compared with the single-pattern chip, it has an additional thin pattern film that is a film containing microchannels for another target pattern. Conventionally, the channel layer, the pattern film, the film, and the storage layer are bonded together. A high-pressure working medium is introduced into the channel of layer B, and the liquid metal will distribute into pattern B. As the pressure is removed, the liquid metal pattern disappears. When the high-pressure working medium is introduced into layer A, the film, together with the pattern film, deforms under high pressure, squeezing the liquid metal to form pattern A. With the pressure decreasing, pattern A disappears. Additionally, multi-layer chips can be designed and fabricated by stacking several films with different microchannels.

There is another way to fabricate a chip to form two patterns rather than fabricating microchannels on a film. Two patterns are located on both sides of the liquid metal storage layer. We designed two antennas; Figure 1c illustrates the structure and working principles. The liquid metal storage layer is made through 3D printing. The cavity is wrapped in the middle, whose top and bottom are bonded with two-channel layers. While the high-pressure working medium is in layer A’s channels, the liquid metal forms pattern A. When the high-pressure working medium (water or air) is in layer B, the film on pattern B compresses the liquid metal to pattern B.

## 3. Materials and Methods

**Figure 2:**
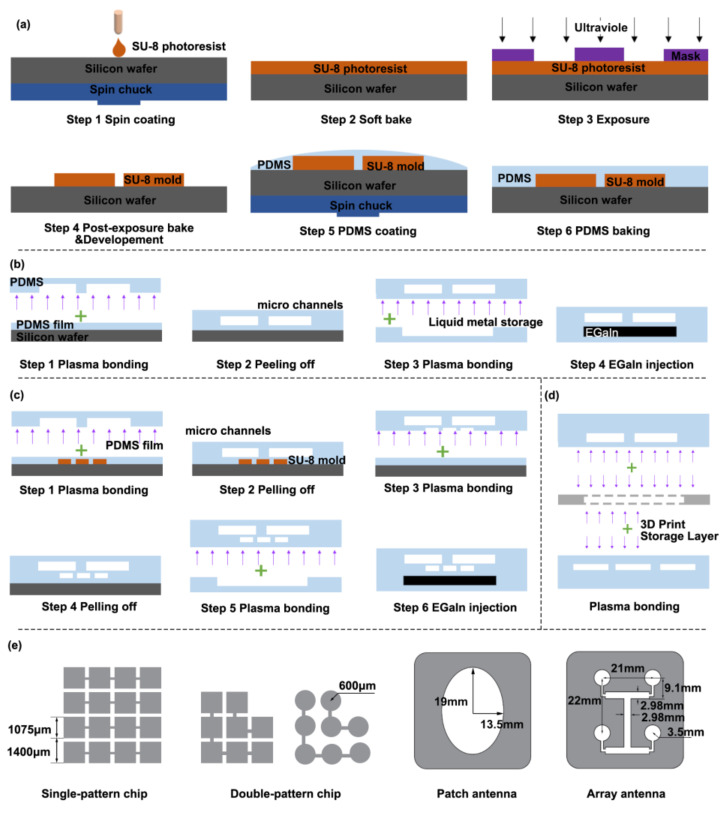
(**a**) Standard soft lithography technology; fabrication process of (**b**) the single-pattern chip; (**c**) the double-pattern chip; (**d**) the reconfigurable antennas; (**e**) and the dimensions of the patterns.

In this work, we use a single-pattern microfluidic chip to test the effect on the patterning results of working medium conditions and chip structure. At the same time, liquid metal patterns in array arrangement are reconfigured in both single-pattern and double-pattern chips. Additionally, two antennas are shown under pressure control. These chips are fabricated with PDMS (dielectric constant εr = 3.7, high loss tan δ = 0.035), and the liquid metal material is a gallium indium alloy (mass fraction Ga = 75.5%, In = 24.5%, conductivity σ = 3.46 ×10^6^ S/m). All channels were obtained through standard soft lithographic technology. First, we need to make an SU-8 mold, as shown in Figure 2a. After spinning and coating the SU-8 photoresist (MicroChem, Austin, TX, USA) on a silicon wafer (Ultrapak^®^ 100 mm, Entegris, Beijing, China) through the steps of pre-baking, exposure, post-baking, and development, the SU-8 mold is obtained on the silicon wafer. Then the PDMS (mixture of base and curing agent at a ratio of 10:1 by weight, Dow Corning, Beijing, China) is poured on the mold, heated at 65 °C for 150 min, and solidified. Finally, the PDMS structure is peeled off. In order to make liquid metal storage layers with different depths, SU8 molds with corresponding heights are made in this paper. The processing parameters are shown in Table 1.

All films are fabricated of PDMS and obtained by a spin-coating process. A blank silicon wafer or a SU-8 mold is placed on the spin coater (EZ4, Lebo Science, Jiangsu, China) to homogenize the PDMS (mixture of base and curing agent at a ratio of 10:1 by weight). Then PDMS is heated at 65 °C for 60 min before solidification. The rotating speed for PDMS homogenizing and other parameters corresponding to different thicknesses of films are shown in Table 2.

The film and other structures are connected by plasma bonding. Figure 2b,c show the fabrication steps of the single-pattern chip and the double-pattern chip. The relevant dimensions are shown in Figure 2c. In each step related to film processing, soaking with anhydrous ethanol for 5 min is conducive to peeling the PDMS layers off the silicon wafers and SU-8 molds easily.

Figure 2d shows the fabrication process of the reconfigurable antennas and their dimensions. The liquid metal storage layer is made by 3D printing technology (Shape1 HD, Rayshape, Suzhou, China). The 3D printing material cannot be directly bonded with the PDMS, and the surface of the 3D printing structure needs to be modified. The 3D printing material is plasma treated in a plasma cleaner for 25 s (YZD08-2C, Yanzhao Technology, Tangshan, China), then it is soaked in APTES solution (5% by volume) at 80 °C for 20 min, then all structures are plasma treated again and bonded in sequence. The dimensions of the patterns are displayed in Figure 2e.

Before the liquid metal is injected into the storage layer, NaOH solution (about 0.5 mL, 0.5 mol/L) is injected into the cavity to prevent the liquid metal from oxidizing and adhering to the PDMS. Liquid metal was injected into the cavity under 500 mbar. Furthermore, the process of injecting was shown in Video S1. A microfluidic flow control device (MFCSTM-EZ, FLUIGENT, Le Kremlin-Bicêtre, France) is used to change the working medium pressure in microchannels. At the same time, a fluorescence microscope (Axio Observer Z1, Carl Zeiss, Oberkochen, Germany) is adopted to monitor the distribution of the liquid metal.

## 4. Results

There are two factors affecting the method of patterning the liquid metal. First, the external control, including the type of working medium and pressure level, determines the pattern. Second, the structure of the chip itself, like the film thickness and the height of the liquid metal cavity channel, may have an influence. This paper adopts the control variate method to discuss the above factors.

### 4.1. Pressure Effect on Patterning

**Figure 3:**
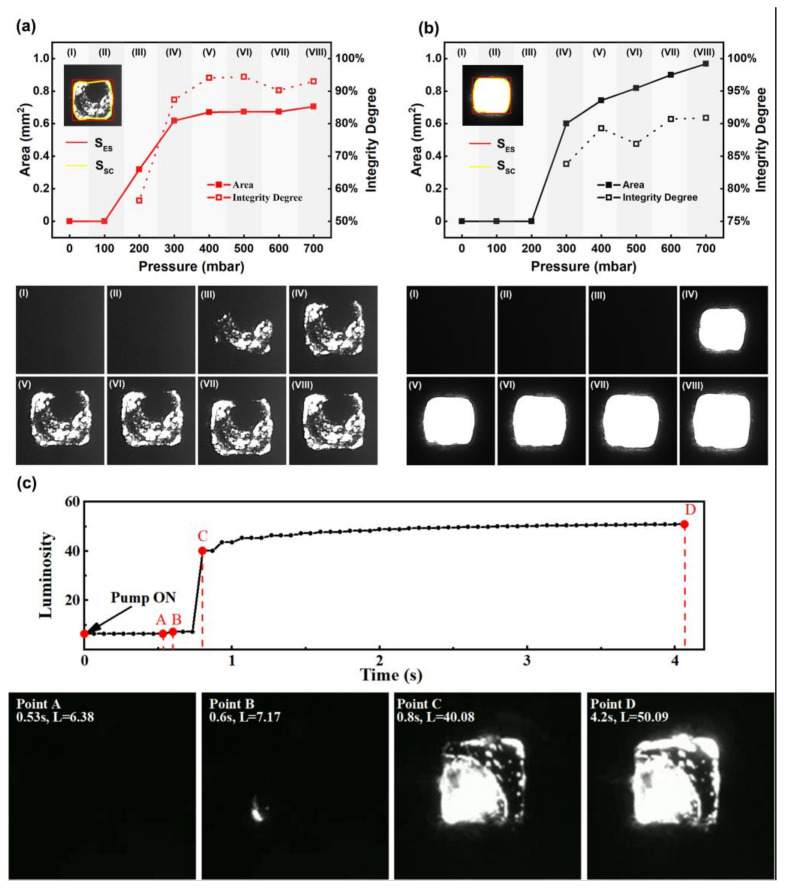
Pressure effect on liquid metal patterning with (**a**) air as working medium and (**b**) water as working medium. I–VIII indicate different pressure applied to the working medium (**c**) The formation process of the liquid metal pattern.

In order to investigate the difference in the liquid metal pattern as the pressure level increases, we recorded the distribution of the patterned liquid metal. In addition, we circumscribed a square around the liquid metal pattern and evaluated the degree of integrity using the Equation (1) as follows:

(1) $$\lambda =\frac{S_{\mathrm{SC}}}{S_{\mathrm{ES}}}\times 100\%$$

where λ is the degree of integrity, $S_{\mathrm{SC}}$ is the area enclosed by splines, and $S_{\mathrm{ES}}$ is the area of the circumscribed square. Liquid metal is injected into a chip with a 22 μm thick film and a 33 μm high cavity. The air performs as the working medium, and the pressure increases by 100 mbar at a time. Figure 3a shows the pictures of liquid metal during the pressure changes from 0 mbar to 700 mbar. Moreover, we held the liquid metal’s edge by splining and calculating the area, as shown in Figure 3a. When air pressure is less than 100 mbar, no pattern appears on the chip. After the air pressure exceeds 300 mbar, the pattern area remains stable at about 0.8 mm^2^. Therefore, the optimal air pressure is 300 mbar.

Using the same experimental process and device, we recorded the changes in the liquid metal when the working medium was changed from air to water. Figure 3b shows the changes under various pressures. If the water pressure is under 200 mbar, there is no pattern in the chip. As the area increases, the film often ruptures when the water pressure reaches 700 mbar. Hence, the maximum water pressure should be 700 mbar; 600 mbar is optimal.

Since water is an incompressible fluid, when it is used as working medium, the area can reach 1 mm^2^, which is much larger than that of compressible air (0.6 mm^2^).

### 4.2. Time Efficiency of the Method

To analyze the formation process of the liquid metal pattern, the pictures were recorded at the rate of 15 frames per second when the working medium was air with a pressure of 600 mbar. During the patterning process, small amount of liquid metal was squeezed aside, and the picture becomes “bright”. The pictures are composed of many pixels, and luminosity, the color depth of pixels, can reflect the brightness with an exact value (ranging from 0 to 255, with 255 for white and 0 for black). We used a MATLAB program (Appendix A) to read the luminosity value and calculate the average value of every frame. Figure 3c shows the entire process. At 0 s, we increased the air pressure from 0 mbar to 600 mbar. After 0.6 s, some liquid metal fractures appeared in the picture, and the luminosity of the frame increased from 6.38 to 7.17. Then at 0.8 s, the pattern was initially formed, and the luminosity reached a value of 40.08. Finally, luminosity data remained stable at 50.09.

### 4.3. Structural Size Effect on Patterning

**Figure 4:**
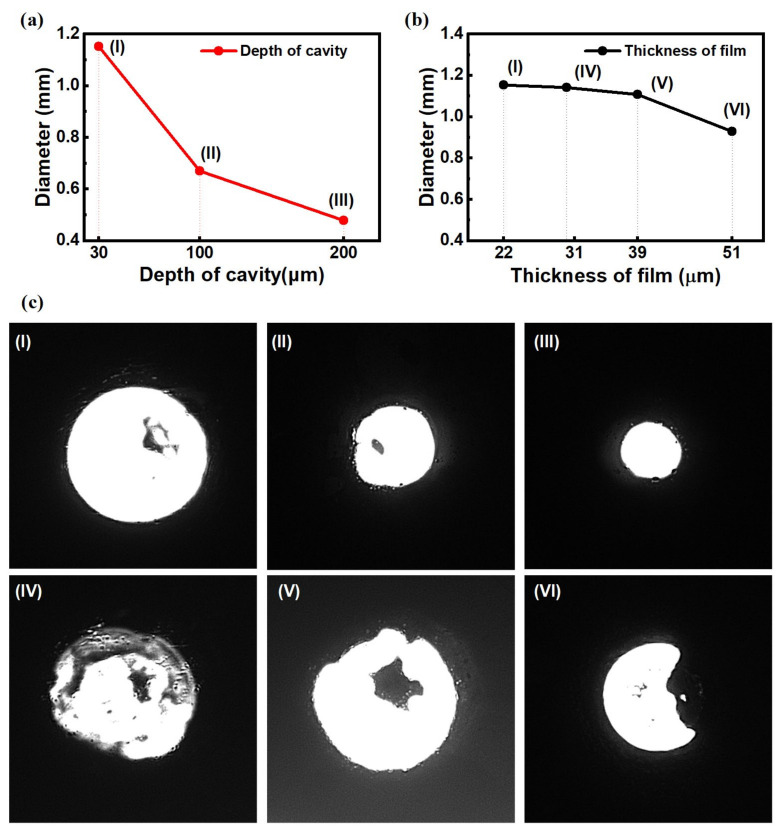
Effect of (**a**) depth of cavity, and (**b**) thickness of films on liquid metal patterning. (**c**) Pictures of liquid metal distribution in different chips. I–VI in (**a**,**b**) correspond to the I–VI in (**c**).

To investigate the difference in the liquid metal pattern in different structures, we use the same experimental process and devices to record the distribution of the liquid metal at 600 mar in chips with different dimensions when the water is used as the working medium. Figure 4a,b show the effect of the depth of cavity and thickness of films on liquid metal patterning. As shown in Figure 4a, when the depth of the cavity increases from 30 μm to 200 μm the diameter of the pattern decreases sharply from 1.15 mm to 0.5 mm. The reason is that the PDMS film will deform in an arch shape at the corners of the cavity; the higher the cavity, the larger the corner effect. Figure 4b shows the effect of film thickness on the patterning. The diameter of the pattern drops from 1.15 mm to 1.0 mm when the film thickness increases from 22 μm to 51 μm. This is because the larger the thickness, the smaller the Young’s modulus of the PDMS film [38]. Thicker films may lead to poorer patterning of liquid metals and even failure to form complete patterns. Correspondingly, pictures of liquid metal distribution in different chips are shown in Figure 4c.

### 4.4. Reconfiguring Test

**Figure 5:**
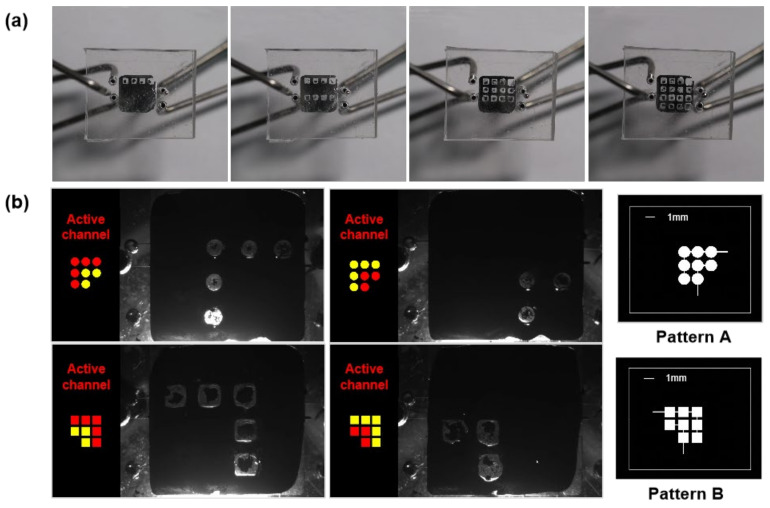
Liquid metal patterns reconfigure in (**a**) a single-pattern chip and (**b**) a double-pattern chip.

When the flow control device instantaneously increases the pressure in channels, the film deforms towards the liquid metal. It occupies the space of the liquid metal in the storage layer. Figure 5a shows a reconfigurable liquid metal array pattern in a single-layer chip. Moreover, Figure 5b shows a double-pattern chip forming more reconfigured patterns; two-patterned dimensions are shown in Figure 5b. As shown in Figure 5b, although the structures of the two patterns are similar, the small blank area in pattern A is circular, and the small blank area in pattern B is square. The complete process is provided in Video S1, which demonstrates that the double-pattern chip can control liquid metal patterning at precise locations and achieve reconfiguration within 1 s. Additionally, this chip enables complex reconfiguration of multiple layers from pattern A to pattern B, which increases the application scenarios.

## 5. Application

**Figure 6:**
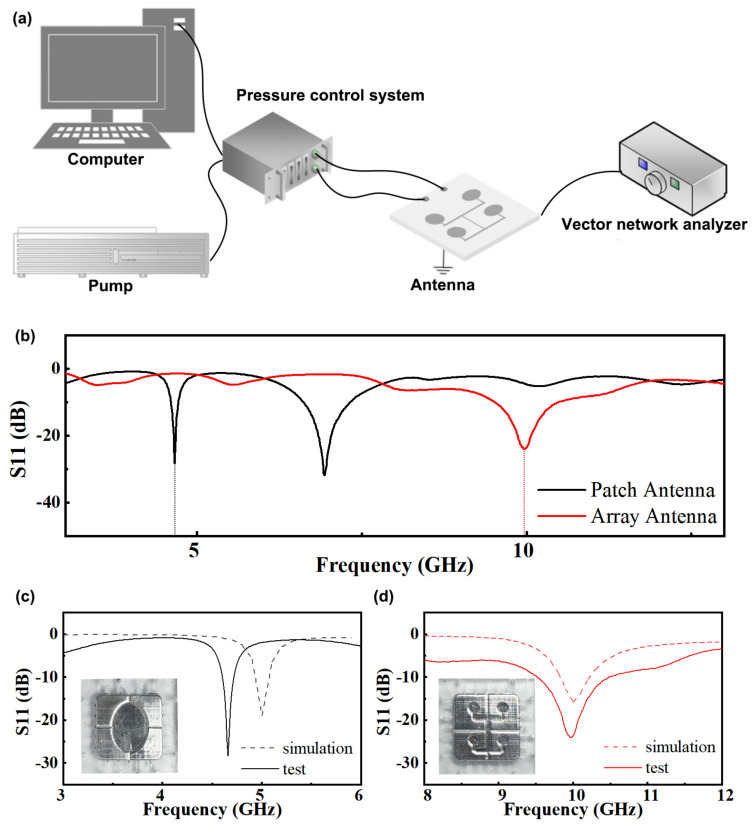
(**a**) Schematic diagram of the reconfigurable antenna control and detection system. (**b**) Reflection coefficients of two antennas during tests. Comparison of simulation and test results in (**c**) 3–6 GHz (**d**) 8–12 GHz.

The array-like single- and double-layer switching of liquid metal patterns provide more diversified application possibilities for the liquid metal in microfluidic devices. A reconfigurable antenna application for fast frequency switching was performed based on this liquid metal patterning switching method. As shown in Figure 6, two types of antenna patterns are designed: the patch antenna and the 2 × 2 array antenna. The detailed geometry size is shown in Figure 2e. The primary electrical parameters, such as response frequency, working frequency band, and bandwidth of both antennas, are simulated using the software ANSYS HFSS. The results are shown in Table 3. The response frequency refers to the correspondence between the extreme gain value; the operating frequency band refers to the frequency range where the gain is less than −10 dB; the bandwidth refers to the width of the operating frequency band; and the relative bandwidth is the ratio of bandwidth to the response frequency.

A vector network analyzer (Keysight Technologies N5247A, Beijing, China) was used to test the gain response of antennas, and the connection between the control device and the detection device of the reconfigurable antenna response frequency is shown in Figure 6a.

The response frequency of the antenna was tested by a vector analyzer, and the results are shown in Figure 6b. Figure 6c,d show the S11, which is the return loss from the antenna in simulation and experiment, respectively, at 3−6 GHz and 8−12 GHz.

Within the band of 3−6 GHz, the corresponding response frequency of the patch antenna is 4.66 GHz, and the bandwidth is 110 MHz. Within the band of 8−12 GHz, the corresponding response frequency of the array antenna is 9.97 GHz, and the bandwidth is 960 MHz.

## 6. Conclusions

This paper proposes a method for controlling liquid metal patterning based on microfluidic technology. This method drives the deformation of the PDMS film by changing the pressure in the microchannels, then the film squeezes the liquid metal in the cavity onto the storage layer, thus rapidly changing the distribution of liquid metal and controlling the formation of different patterns of liquid metal on a two-dimensional scale.

In addition, this paper analyzes a variety of factors that affect patterning in detail. As for compressible working mediums, such as air, the deformation control effect is not as good as that of incompressible fluids, such as water. Furthermore, the pattern size of liquid metal is closely related to the channel pressure. As the pressure increases, the effect of liquid metal patterns will gradually grow. After the pressure increases to a certain extent, the pattern performance gradually remains stable. Meanwhile, as the thickness of PDMS films and the height of the storage layers increase, the patterning performance worsens. The above conclusions can provide guidelines for the design and fabrication of reconfigurable liquid-metal-based microfluidic chips.

An array switch and a two-layer switch of liquid metal patterns were created using this method. Moreover, we propose a reconfigurable antenna whose harmonic frequencies vary under various working states and can meet an array of needs. This paper offers a promising method for flexible sensors, wearable devices, and other applications.

## 7. Patents

The Chinese invention patent based on this work has been authorized (ZL201910566954.1).

## Figures and Tables

**Table 1:** Fabrication parameters of SU-8 mold.

Height of SU-8 Mold (μm)	SU-8 Series	Spin-Coating Speed (rpm)	Exposure Time (s)
30	2050	3000	8
100	2075	1500	10
200	2075	1000	12

**Table 2:** Fabrication parameters of PDMS film.

PDMS Film Thickness (μm)	Spin-Coating Speed (rpm)	Spin-Coating Time (min)
22	3000	2
31	2500	2
39	2000	2
51	1500	2

**Table 3:** Basic electrical parameters of two antennas from simulation.

Type Frequency (GHz)	Frequency (GHz)	Operating Frequency Band (GHz)	Bandwidth (MHz)
Patch antenna	5	4.93−5.07	140
Array antenna	10	9.83−10.22	390

## Data Availability

The data presented in this study are available on request from the corresponding author. The data are not publicly available due to the project requirement.

## Supplementary Materials

The following supporting information can be downloaded at: https://www.mdpi.com/article/10.3390/mi14040717/s1, Video S1.

## Appendix A

Matlab program for luminosity
img_origin = imread(‘xxx.png); pc_gray = rgb2gray(jpg); I_db = double(pc_gray);
